# VP2 of Chicken Anaemia Virus Interacts with Apoptin for Down-regulation of Apoptosis through De-phosphorylated Threonine 108 on Apoptin

**DOI:** 10.1038/s41598-017-14558-8

**Published:** 2017-11-01

**Authors:** Guan-Hua Lai, Yi-Yang Lien, Ming-Kuem Lin, Jai-Hong Cheng, Jason TC Tzen, Fang-Chun Sun, Meng-Shiunn Lee, Hsi-Jien Chen, Meng-Shiou Lee

**Affiliations:** 1 Graduate Institute of Biotechnology, National Chung Hsing University, Taichung, 40402 Taiwan; 2 Department of Veterinary Medicine, National Pingtung University of Science and Technology, Pingtung, 91201 Taiwan; 3 Department of Chinese Pharmaceutical Science and Chinese Medicine Resources, China Medical University, Taichung, 40402 Taiwan; 4 Center for Shockwave Medicine and Tissue Engineering, Department of Medical Research, Kaohsiung Chang Gung Memorial Hospital and Chang Gung University College of Medicine, Kaohsiung, 83301 Taiwan; 5 Department of Bioresources, Da-Yeh University, Changhua, 515 Taiwan; 6 Research Assistance Center, Show Chwan Memorial Hospital, Changhua, 500 Taiwan; 7 Department of Safety, Health and Environmental Engineering, Ming Chi University of Technology, New Taipei, 24301 Taiwan

## Abstract

Chicken anaemia virus (CAV) is an important contagious agent that causes immunosuppressive disease in chickens. CAV Apoptin is a nucleoplasmic shuffling protein that induces apoptosis in chicken lymphoblastoid cells. In the present study, confocal microscopy revealed co-localisation of expressed CAV non-structural protein VP2 with Apoptin in the nucleus of MDCC-MSB1 cells and the nucleoplasmic compartment of CHO-K1 cells. *In vitro* pull-down and *ex vivo* biomolecular fluorescent complementation (BiFC) assays further showed that the VP2 protein directly interacts with Apoptin. Transient co-expression of VP2 and Apoptin in MDCC-MSB1 cells significantly decreased the rate of apoptosis compared with that in cells transfected with the Apoptin gene alone. In addition, the phosphorylation status of threonine 108 (Thr108) of Apoptin was found to decrease upon interaction with VP2. Although dephosphorylated Thr108 did not alter the subcellular distribution of Apoptin in the nucleus of MDCC-MSB1 cells, it did suppress apoptosis. These findings provide the first evidence that VP2 directly interacts with Apoptin in the nucleus to down-regulate apoptosis through alterations in the phosphorylation status of the latter. This information will be useful to further elucidate the underlying mechanism of viral replication in the CAV life cycle.

## Introduction

Chicken anaemia virus (CAV) is a small, icosahedral and non-enveloped virus belonging to genus *Gyrovirus* of the *Anelloviridae* family^1^. Its genome is a circular single-stranded DNA molecule of approximately 2.3 kb^2^. CAV reportedly only infects chickens. The CAV virion is resistant to harsh environmental conditions, such as solvent, disinfectant, thermal, acidic or alkaline treatments, and the virus is distributed worldwide^3^. In addition, CAV is highly infectious to young chickens, particularly those less than 2 weeks old^4^. CAV infections typically lead to bone marrow aplasia and lymphoid tissue destruction in young chickens^3,5^, resulting in severe anaemia, lymph organ atrophy and immunosuppression^2,3,5^. Moreover, the immunosuppression resulting from CAV typically causes serious secondary infections by other viruses and even decreases the efficacy of vaccines after immunisation^2^. Therefore, CAV is considered to be an economically important virus to the poultry industry.

During the CAV life cycle, three viral proteins, VP1, VP2 and VP3, are translated in the host from the unspliced mRNA^2,6^. The VP1 protein (51 kDa) is the sole structural protein responsible for the assembly of virus particles^2,7^. A non-structural protein, VP2 (24 kD) not only possesses dual-specificity phosphatase activity but also functions as a scaffold for capsid assembly^7,8^. Lastly, VP3, also called “Apoptin”, is the smallest viral protein of CAV (13 kD), and exhibits apoptosis-inducing activity in specific cells^9^.

Apoptosis often occurs in virus-infected cells. In the early stages of virus infection, host cells trigger the process of apoptosis as a defence against viral infection, inducing an immune response to attenuate virus spread^10^. To establish a productive infection, specific viral proteins produced during replication suppress the induction of apoptosis in host cells^9^. For example, human adenovirus proteins E1b 55 K and E1b 19 K suppress p53-dependent or p53-independent apoptosis^10–12^. Similarly, the caspase inhibitor p35 of baculovirus down-regulates apoptosis^9,12,13^. Upon propagation of progeny viruses in host cells, up-regulation of apoptosis is induced by viral proteins, such as the non-structural proteins NS4a and NS3 of Hepatitis C virus, which decrease mitochondrial membrane potential and increase caspase-8 activity, respectively, to induce apoptosis during the late stages of viral replication^14–16^.

During CAV infection, apoptosis in thymocytes caused by Apoptin of CAV is considered to be a major clinical symptom attributable to the main pathological mechanism in the host^9^. Apoptin possesses tumour-specific apoptosis-inducing activity, thereby attracting much attention for use in medical applications^17^. The apoptotic mechanism of Apoptin in cancer cells has been widely discussed in previous studies^18–22^. For instance, the phosphorylation status of threonine 108 (Thr108) on Apoptin has a key role in triggering apoptosis in cancer cells^23^, and the subcellular distribution of Apoptin is altered from the nucleus to the nucleoplasmic compartment upon Thr108 dephosphorylation^23^. However, the mechanism by which dephosphorylation of Apoptin Thr108 exerts its effects remains elusive.

VP2 and Apoptin are expressed at higher levels than is VP1 during the first 12–24 hrs post-infection with CAV^24^. VP2 and Apoptin also show a characteristic nuclear localisation^22,25^. Mutagenesis of residues of the catalytic site of VP2 not only significantly decrease viral replication and pathogenicity but also alter the subcellular distribution of Apoptin along with nucleoplasmic shuffling of Apoptin showed^8^. Taken together, these findings suggest that interaction between VP2 and Apoptin occurs under certain conditions, though further investigation is needed. Thus, to further clarify the role of viral proteins such as VP2 and Apoptin in the life cycle of CAV, protein-protein interaction between VP2 and Apoptin and the effect of the phosphatase activity of VP2 on apoptosis triggered by apoptin were investigated and verified in the present study.

## Results

### Co-localisation of VP2 and Apoptin in cells

**Figure 1:**
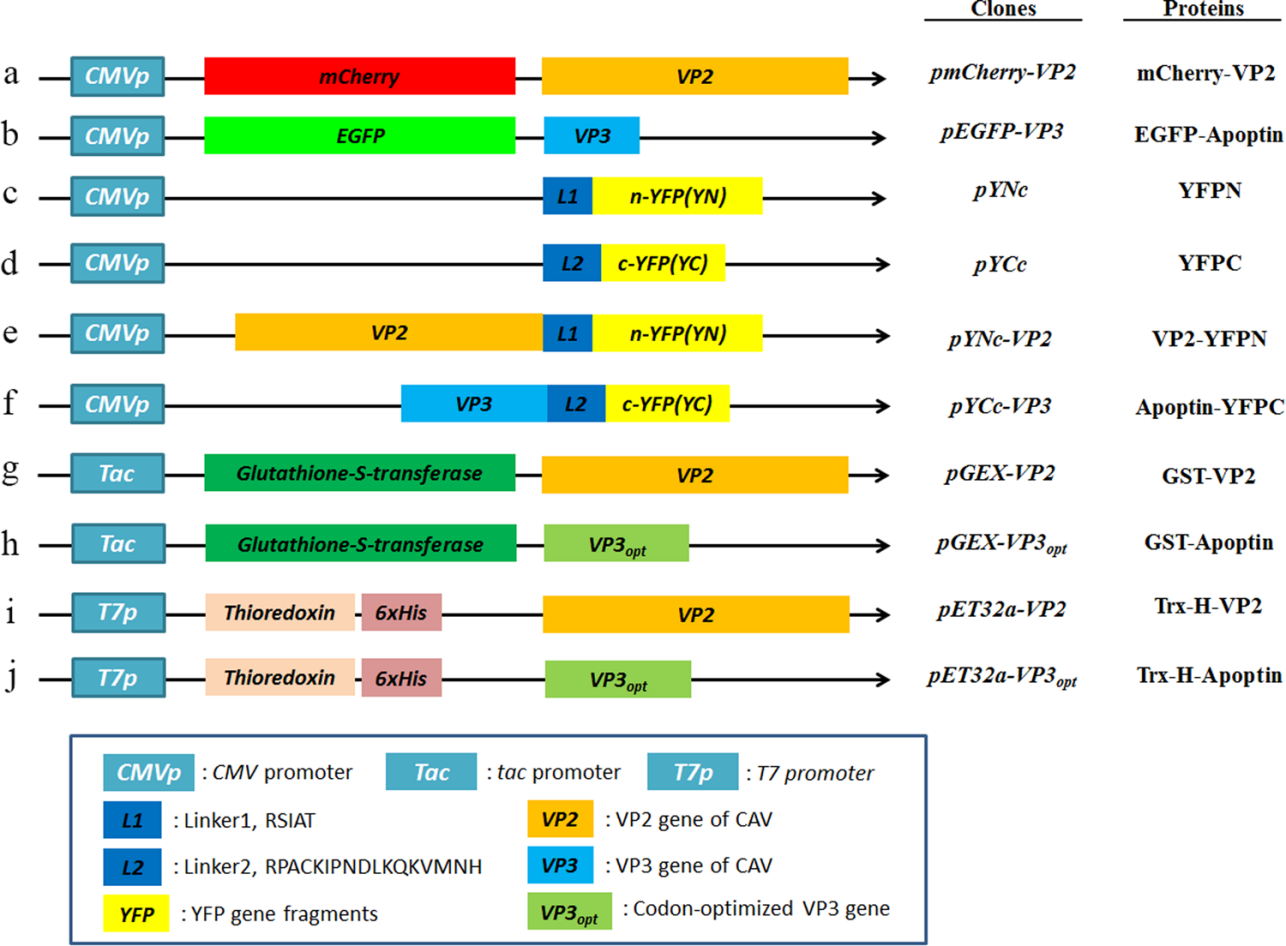
Schematic diagram of the constructs used for expression of VP2 and Apoptin of CAV. Constructs a and b used for investigation of the subcellular distribution of VP2 and Apoptin are indicated as the VP2 gene and VP3 gene, which were fused with fluorescent mCherry and EGFP, respectively, in the transient expression vectors pmCherry-C1 and pEGFP-C2 to generate the recombinant plasmids pmCherry-VP2 and pEGFP-VP3, respectively. Constructs c and d were used as negative controls in *ex vivo* BiFC assays and indicate that the two gene fragments of YFP encoding the N-terminal domain (YN, residues 1–154) and C-terminal domain (YC, residues 155–238) were respectively cloned into plasmid pcDNA3.1 for cell transfection. Linker 1 (L1) and linker 2 (L2) represent sequences encoding two small peptides (RSIAT for L1 and RPACKIPNDLKQKVMNH for L2) used for the linkage of YN and YC. The VP2 and VP3 genes were respectively fused to the sequences of YN and YC using L1 and L2 as linkers, resulting in constructs e and f. Constructs g, h, i and j were used for *in vitro* GST pull-down assays and were generated for expression of VP2 and Apoptin of CAV by fusion of the VP2 and VP3 genes. Constructs g and h were generated from pGEX-4T-1 for GST fusion protein expression. The thioredoxin-His (Trx-H) tag for VP2 and apoptin was obtained via expression of the Trx-H coding sequence from plasmid pET32a, illustrated as constructs i and j, respectively.

**Figure 2:**
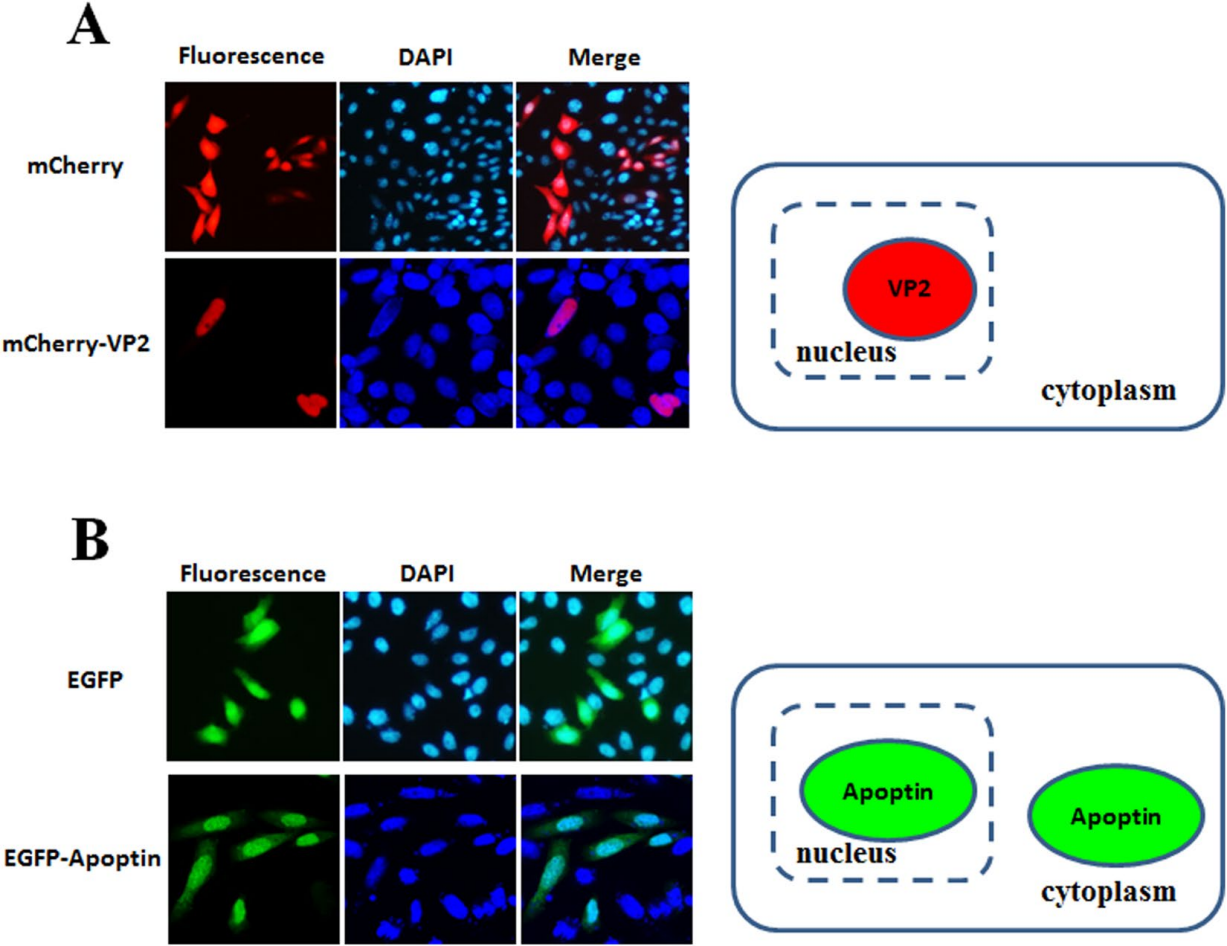
Subcellular localisation of CAV VP2 and Apoptin in CHO-K1 cells. CHO-K1 cells were transfected with pmCherry-C1 and pmCherry-VP2 (**A**) or pEGFP-C2 and pEGFP-VP3 (**B**), respectively. At 48 hrs post-transfection, the cells were fixed and stained with DAPI as a nuclear indicator, followed by confocal microscopy. The distribution of red fluorescence in CHO-K1 cells indicated the subcellular localisation of overexpressed mCherry or mCherry-VP2; green fluorescent indicated the localisation of EGFP or EGFP-Apoptin. The localisation of VP2 and Apoptin in CHO-K1 cells was determined after comparing the merged images between the sole fluorescent protein and fluorescent protein-fused protein.

**Figure 3:**
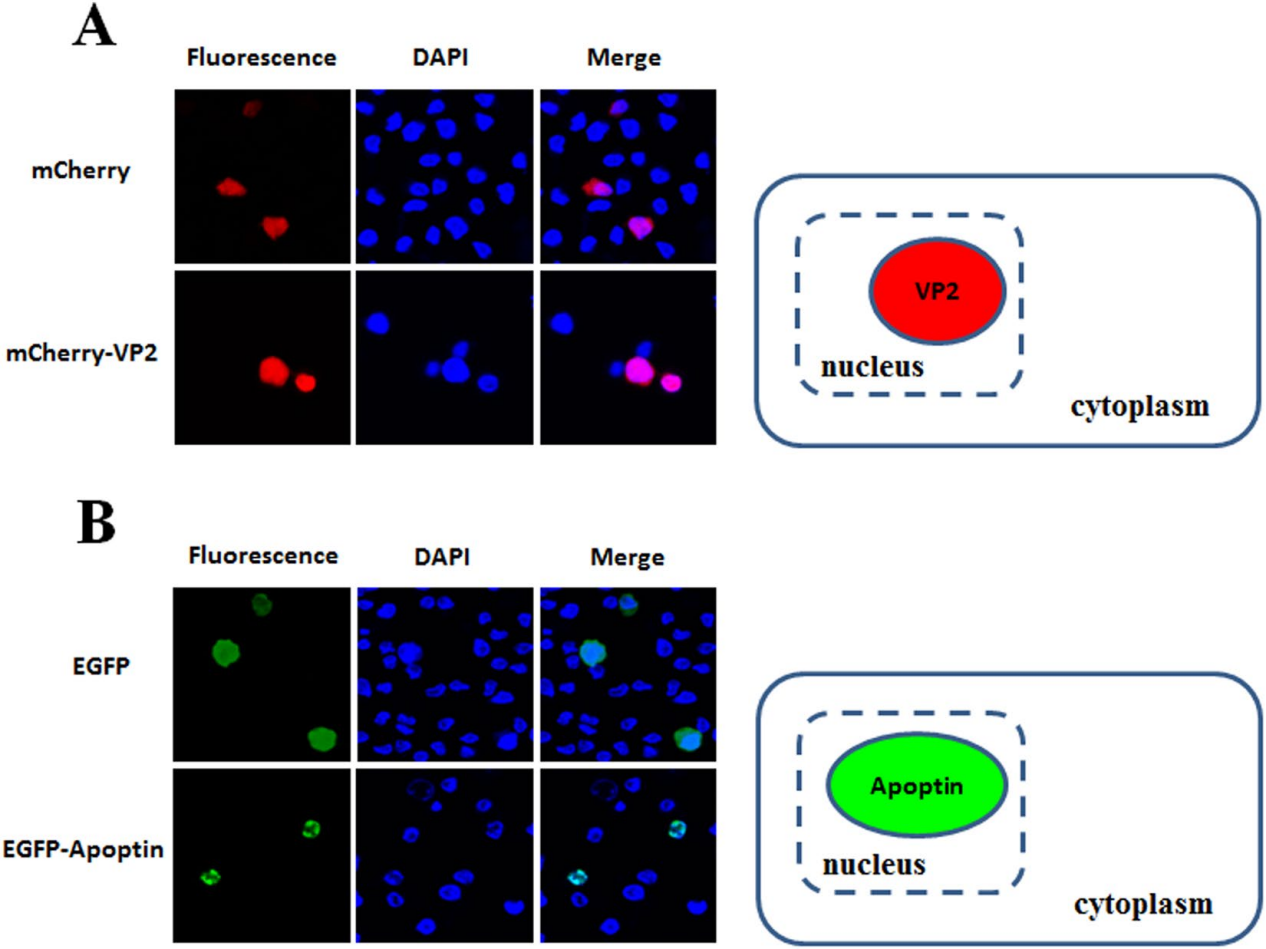
Nuclear localisation of VP2 and Apoptin in MDCC-MSB1 cells. At 48 hrs post-transfection with pmCherry-C1 and pmCherry-VP2 (**A**) or pEGFP-C2 and pEGFP-VP3 (**B**) via electroporation, MDCC-MSB1 cells were fixed and stained with DAPI. The distribution of mCherry and mCherry-VP2 (red), EGFP and EGFP-Apoptin (green) was examined by confocal fluorescence microscopy to determine the subcellular localisation of the recombinant proteins.

**Figure 4:**
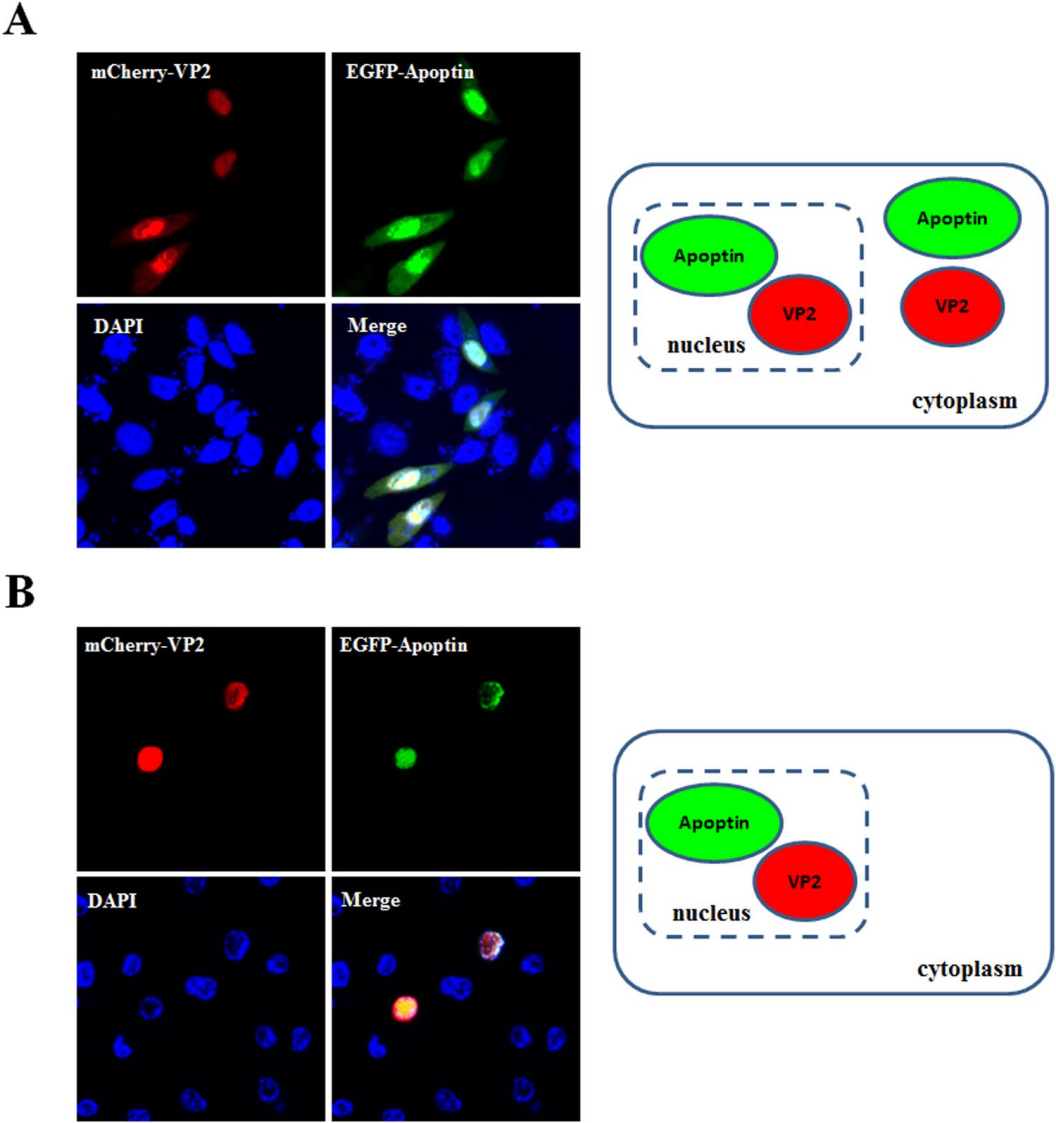
Co-localisation of VP2 and Apoptin in cells. CHO-K1 (**A**) and MDCC-MSB1 (**B**) cells were both co-transfected with mCherry-VP2 (red) and EGFP-Apoptin (green)-expressing plasmids. At 48 hrs post-transfection, the cells were fixed, and the subcellular localisation of VP2 and Apoptin was directly analysed by confocal fluorescence microscopy. The transfected cells were also stained with DAPI to reveal the nucleus.

The distribution of proteins to the same subcellular compartment increases the potential and likelihood of protein-protein interactions. Thus, to confirm interaction between VP2 and Apoptin, the localisation of CAV VP2 and Apoptin proteins was examined. First, respective recombinant plasmids pmCherry-C1 and pEGFP-C2 carrying sequences encoding the fluorescent tags mCherry and enhanced green fluorescent protein (EGFP) fused to the VP2 and VP3 genes were prepared and transfected into CHO-K1 cells (Fig. 1, constructs a and b). At 48 hrs post-transfection, red and green fluorescence observation demonstrated expression of mCherry-VP2 and EGFP-Apoptin, respectively (Fig. 2A and B). Further examination of the subcellular localisation of the fluorescent fusion proteins revealed significant accumulation of mCherry-VP2 and EGFP-Apoptin in the nucleus of CHO-K1 cells. EGFP-Apoptin was not only detected in the nucleus but also in the cytoplasmic compartments of CHO-K1 cells (Fig. 2B), yet both mCherry-VP2 and EGFP-Apoptin were only observed in the nucleus of MDCC-MSB1 cells (Fig. 3A and B). Both fluorescent proteins were found only in the nucleus of MDCC-MSB1 cells upon co-expression (Fig. 4B), revealing no differences in localisation and paralleling the expression patterns observed when mCherry-VP2 and EGFP-Apoptin were expressed alone in these cells. In contrast, co-expressed mCherry-VP2 and EGFP-Apoptin not only localised to the cytosol but also to the nucleus of CHO-K1 cells (Fig. 4A). In particular, regardless of whether it was expressed alone or with mCherry-VP2, the nucleoplasmic localisation of EGFP-Apoptin was not altered. However, when mCherry-VP2 was co-expressed with EGFP-Apoptin in CHO-K1 cells, the subcellular localisation of the former was significantly different compared with when it was expressed alone in these cells (Fig. 4A). Because the VP2 protein lacks a nuclear export signal (NES)^25^, the observed fluorescence of mCherry-VP2 simultaneously in the nucleus and cytosol of CHO-K1 cells might reflect interaction of VP2 with Apoptin, a possibility that requires further evidence.

### Identification of the interaction between VP2 and Apoptin using biomolecular fluorescence complementation (BiFC) and GST pull-down assays

**Figure 5:**
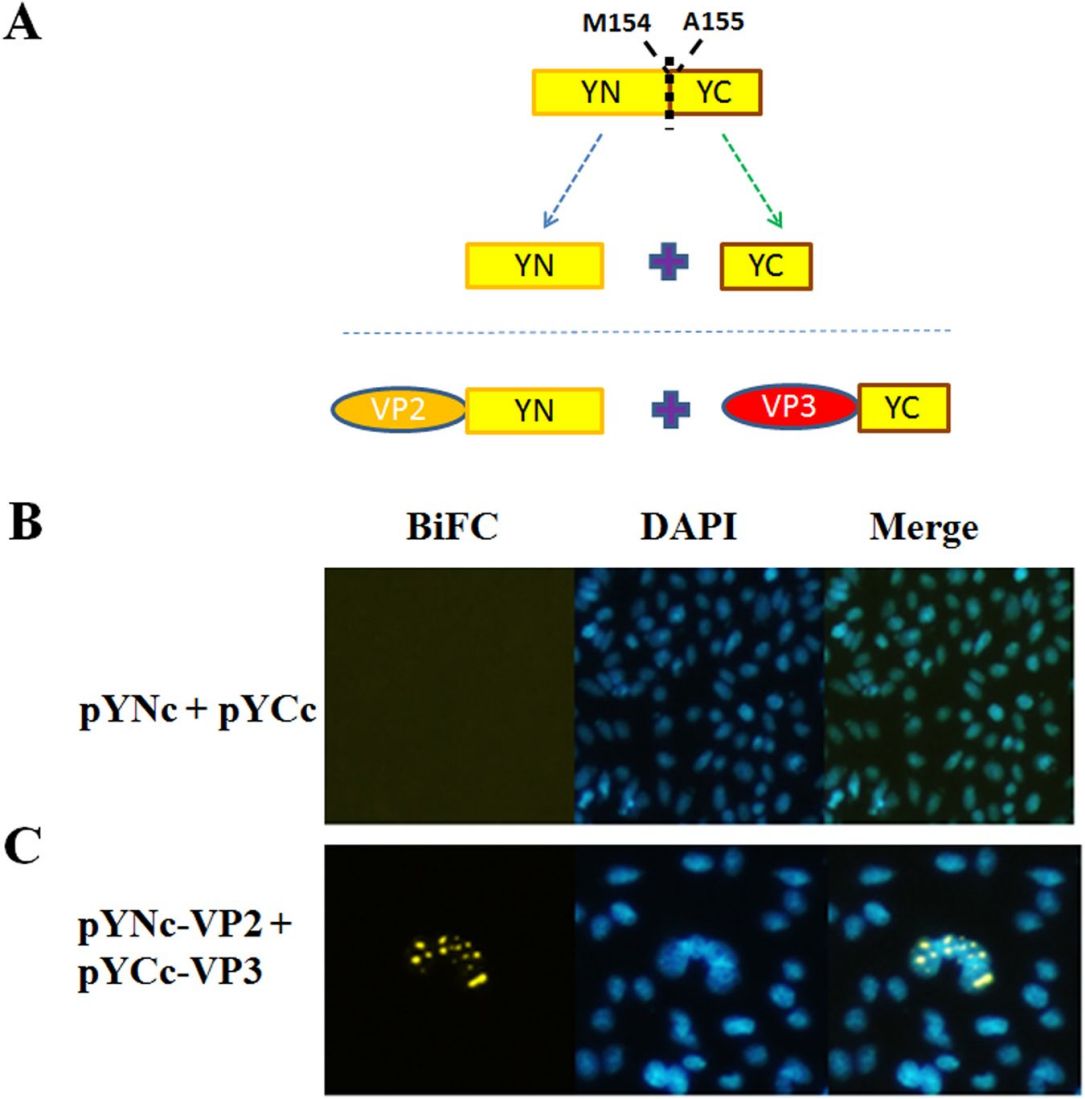
Identification of the interaction between VP2 and Apoptin using *ex vivo* BiFC assays. Two YFP gene fragments, YN and YC, were generated from amino acid residues of 154-155 split YFP and fused to the C-terminus of CAV VP2 and VP3 gene, respectively, to generate recombinant constructs for the BiFC assay (**A**). After co-expression of the two constructs in CHO-K1 cells for 48 hrs, the cells were fixed and stained with DAPI to reveal nuclear localisation. The distribution of yellow fluorescence, indicating BiFC, was analysed by confocal fluorescence microscopy (**B**). The BiFC signal indicated the protein-protein interaction between VP2 and Apoptin, and this interaction was primarily located in the nucleus.

**Figure 6:**
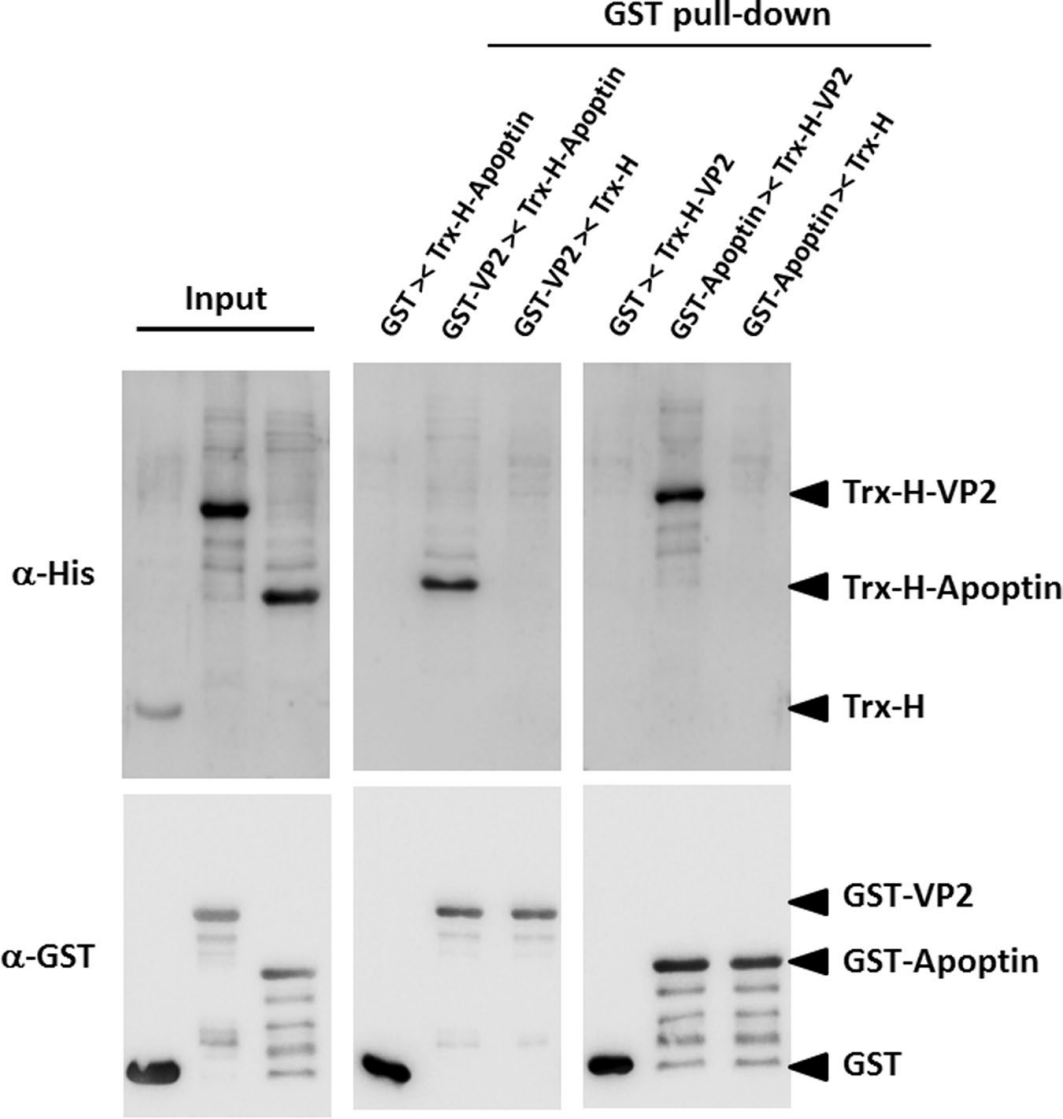
Identification of the VP2-Apoptin interaction using an *in vitro* GST pull-down assay. Recombinant GST, GST-VP2 or GST-Apoptin were immobilised on glutathione-Sepharose resin and incubated with purified Trx-H, Trx-H-VP2 or Trx-H-Apoptin protein. After pull-down of the bound protein, the co-eluted fractions were separated by SDS-PAGE and analysed by western blotting with anti-GST or anti-6xHis monoclonal antibodies. The input panel shows the relative probed signals of recombinant proteins used in this assay. The GST pull-down panel indicates the fraction of co-elution with GST-fused protein (bait) and corresponding Trx-H-fused protein (prey).

An *ex vivo* BiFC assay was performed to verify the interaction between VP2 and Apoptin. Two recombinant plasmids were constructed, as illustrated in Fig. 1. The recombinant plasmid pYNc-VP2 harbours the VP2 gene fused with the N-terminal domain of yellow fluorescent protein (YFP) at its C-terminus. The C-terminal domain of YFP was also fused to the C-terminus of apoptin, resulting in the pYCc-VP3 plasmid (Fig. 5A). When the plasmids pYNc-VP2 and pYCc-VP3 were co-transfected into CHO-K1 cells, emission of YFP fluorescence was observed in the 4′,6-diamidino-2-phenylindole (DAPI)-stained nucleus (Fig. 5C). In contrast to the control, co-transfection of null plasmids separately carrying the N-terminal and C-terminal domains of YFP did not result in YFP emission in the nucleus of CHO-K1 cells after laser excitation (Fig. 5B). Thus, the BiFC assay results reveal interaction between VP2 and Apoptin. To further confirm the VP2-Apoptin interaction determined by the BiFC assay, *in vitro* glutathione S-transferase (GST) pull-down assays were performed (Fig. 6). When Ni^2+^-nitrilotriacetic acid (NTA) column-purified Apoptin containing a thioredoxin-His tag (Trx-H-Apoptin) was loaded onto a GST affinity column, Trx-H-Apoptin was co-eluted with GST-fused VP2 (GST-VP2), as illustrated by the western blot analysis shown in Fig. 6. Similarly, when GST-fused Apoptin (GST-Apoptin) was purified using a GST affinity column, thioredoxin-His-fused VP2 (Trx-H-VP2), which was reloaded onto the same column, was also co-purified. In contrast to the control, thioredoxin-His was not pulled down by either GST-VP2 or GST-Apoptin using the GST affinity column; in addition, Trx-H-Apoptin or Trx-H-VP2 was not pulled down using GST. Taken together, the protein-protein interaction between VP2 and Apoptin was identified and confirmed by BiFC and GST pull-down assays.

### VP2 down-regulates the apoptosis triggered by Apoptin

**Figure 7:**
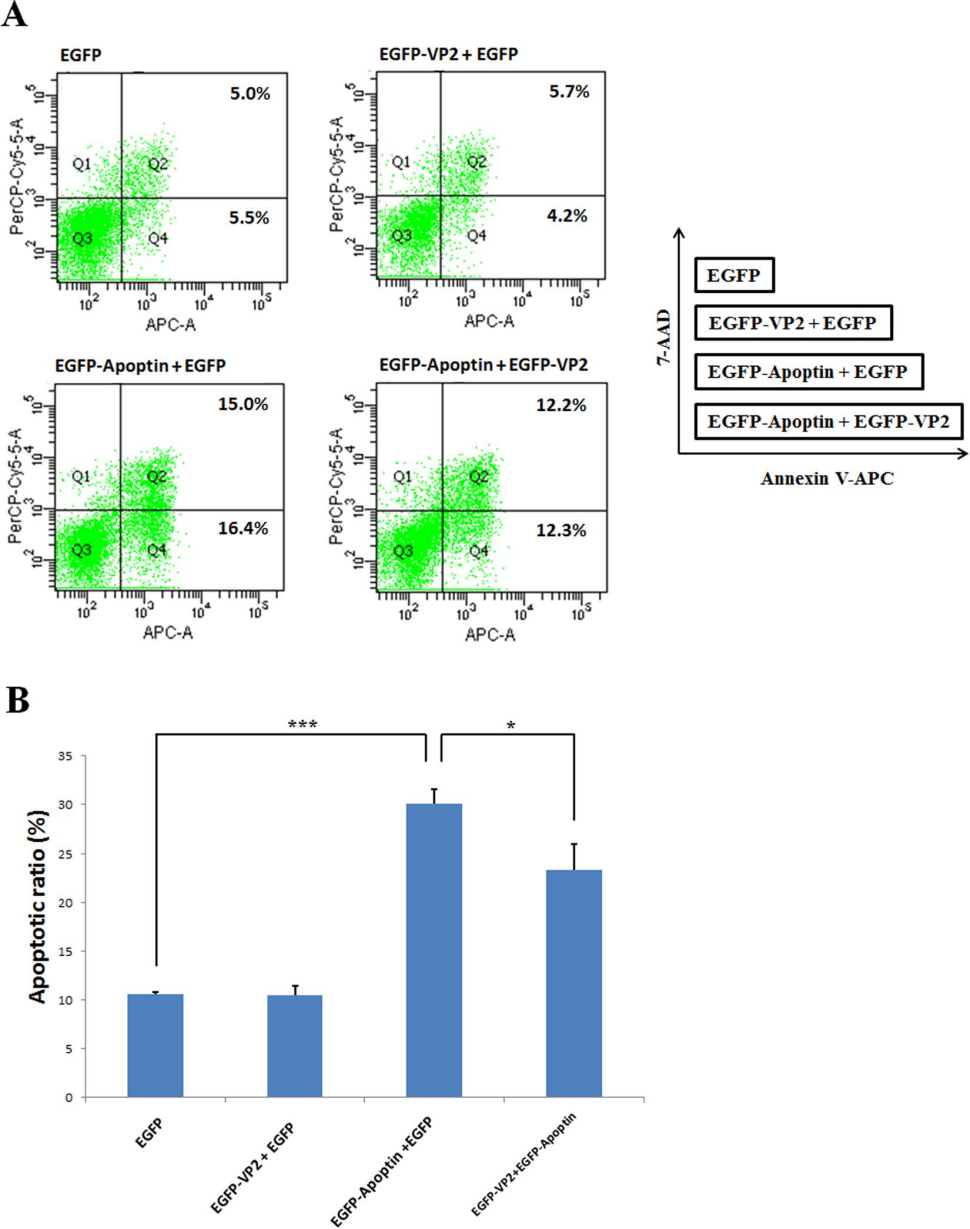
The apoptosis-inducing ability of Apoptin was reduced by VP2. MDCC-MSB1 cells were transfected with different expression plasmids. The corresponding co-transfected plasmids are shown in the diagram in the right panel. FACS dot plots of Annexin-V APC (x-axis) versus 7-AAD (y-axis) were obtained at 2 days post-transfection, showing different populations of early apoptotic cells from late apoptotic and necrotic cells. The Q4 quadrant represents early apoptotic cells (Annexin-V positive); the Q2 quadrant indicates late and necrotic cells (Annexin-V and 7-AAD positive) (**A**). Bar diagrams showing the average counts of triplicate results of apoptotic assays analysed by flow cytometry (**B**). Error bars indicate the standard deviation of three independent experiments. The levels of apoptotic ratios under different conditions were significantly different according to ANOVA analysis. ***p < 0.001; *p < 0.05.

Previous studies have shown that Apoptin has apoptosis-inducing activity in many cancer cell lines, including chicken lymphoblastoid MDCC-MSB1 cells^9,17^. As described above, the protein-protein interaction between VP2 and Apoptin was confirmed using BiFC and GST pull-down assays. To assess the effect of VP2-Apoptin interaction on apoptosis, apoptin-induced apoptosis was further examined in MDCC-MSB1 cells co-transfected with plasmids carrying the VP2 gene (pEGFP-VP2) and the Apoptin gene (pEGFP-VP3). The results of flow cytometry analysis, illustrated in Fig. 7A, showed that co-transfection of pEGFP-VP2 and pEGFP-VP3 in MDCC-MSB1 cells significantly decreased the apoptosis rate compared with co-transfection of pEGFP and pEGFP-VP3. The rate of apoptosis concurrently decreased in the early (quadrant 4, Q4) and late (quadrant 2, Q2) phases of apoptosis. In contrast to pEGFP or pEGFP-VP2 alone, GFP or VP2 alone did not trigger apoptosis in MDCC-MSB1 cells. There were no differences in apoptosis among above treatments. The histogram in Fig. 7B shows that the apoptosis induced by Apoptin was markedly reduced by approximately 7% (*p* < 0.05), from 31% to 24%, when VP2 was expressed in these cells. These results demonstrate that VP2 might play a role in suppressing the apoptosis triggered by Apoptin in terms via their interaction.

### Dephosphorylation of Thr108 on Apoptin by VP2 attenuates apoptosis

**Figure 8:**
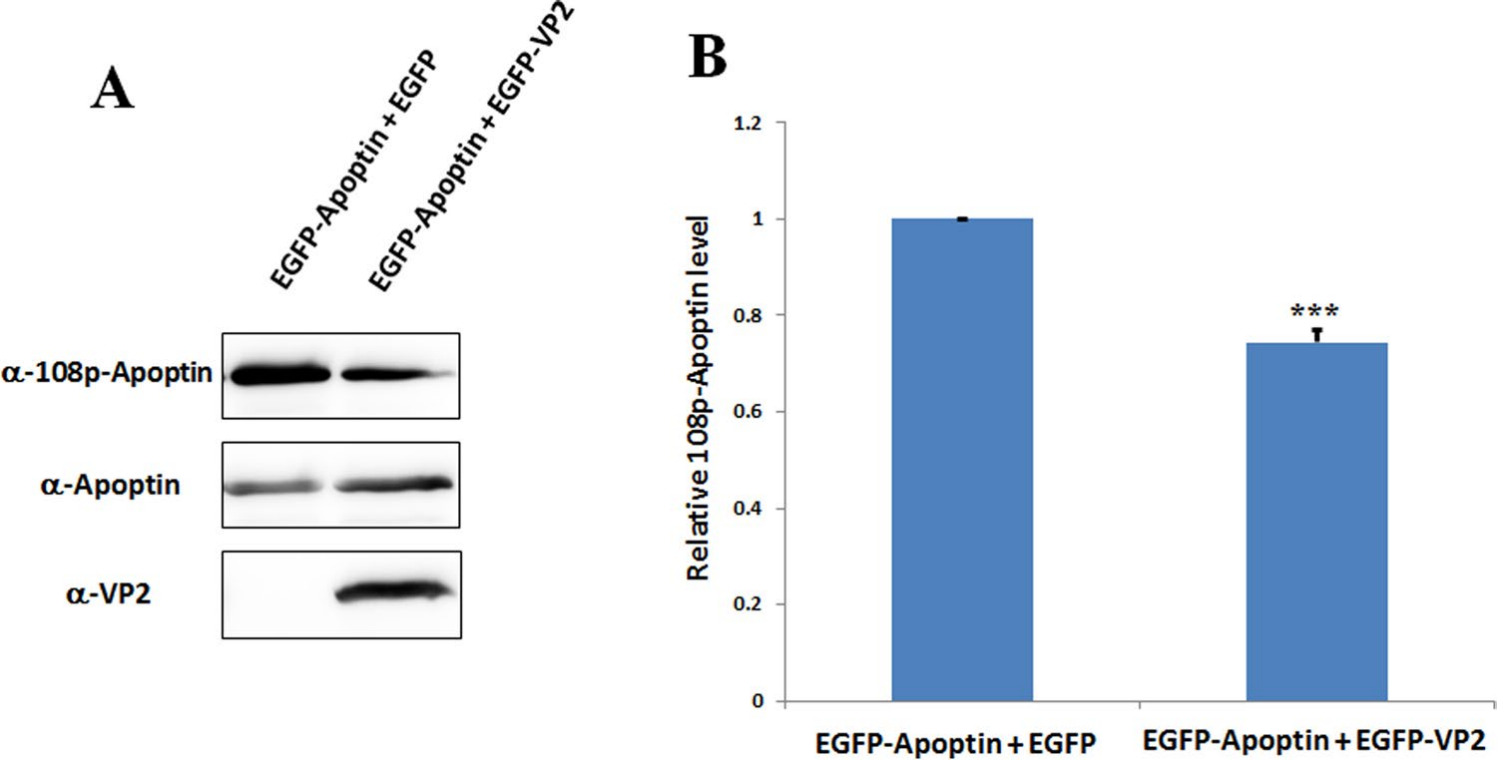
Phosphorylation of Apoptin Thr108 was reduced by VP2. Co-transfected MDCC-MSB1 cells were collected, and the phosphorylation status of Apoptin was analysed by western blotting using polyclonal anti-108p-Apoptin antibodies. Expression of VP2 and Apoptin was also detected using the corresponding antibodies (**A**). After quantifying the image intensity using ImageJ software, the results are represented as a bar diagram **(B)** showing the average counts of triplicate results. ***p < 0.001 significant difference vs. co-expression of EGFP-Apoptin and EGFP.

Thr108 of Apoptin is an essential residue for inducing apoptosis^23^, and VP2 phosphatase activity has been demonstrated in a previous study^8^. Thus, to confirm whether the phosphorylation status of Apoptin Thr108 is altered during the interaction of VP2 with Apoptin, antibodies specific for phosphorylated Thr108 were used to evaluate its phosphorylation status when VP2 was simultaneously expressed with Apoptin in cells. As depicted in Fig. 8A, expression of Apoptin alone or VP2 and Apoptin was detected in MDCC-MSB1 cell extracts by western blot analysis using anti-VP2 and anti-Apoptin antibodies, respectively. Similarly, phosphorylation of Apoptin Thr108 was also detected when transfection of Apoptin alone or VP2 and Apoptin was performed (Fig. 8A). However, the phosphorylation level of Thr108 on Apoptin differed slightly when detected using anti-phosphorylated Apoptin antibodies. After normalisation of the Apoptin protein level, as shown in Fig. 8B, compared with Apoptin treatment alone, the relative phosphorylation level of Apoptin was found to be markedly reduced, by approximately 25.6%. This result demonstrates that the interaction between VP2 and Apoptin results in the attenuation of apoptosis through dephosphorylation of Apoptin Thr108. Importantly, VP2 plays a key role in the dephosphorylation of Apoptin.

### Dephosphorylation of Thr108 does not affect the subcellular distribution of Apoptin

**Figure 9:**
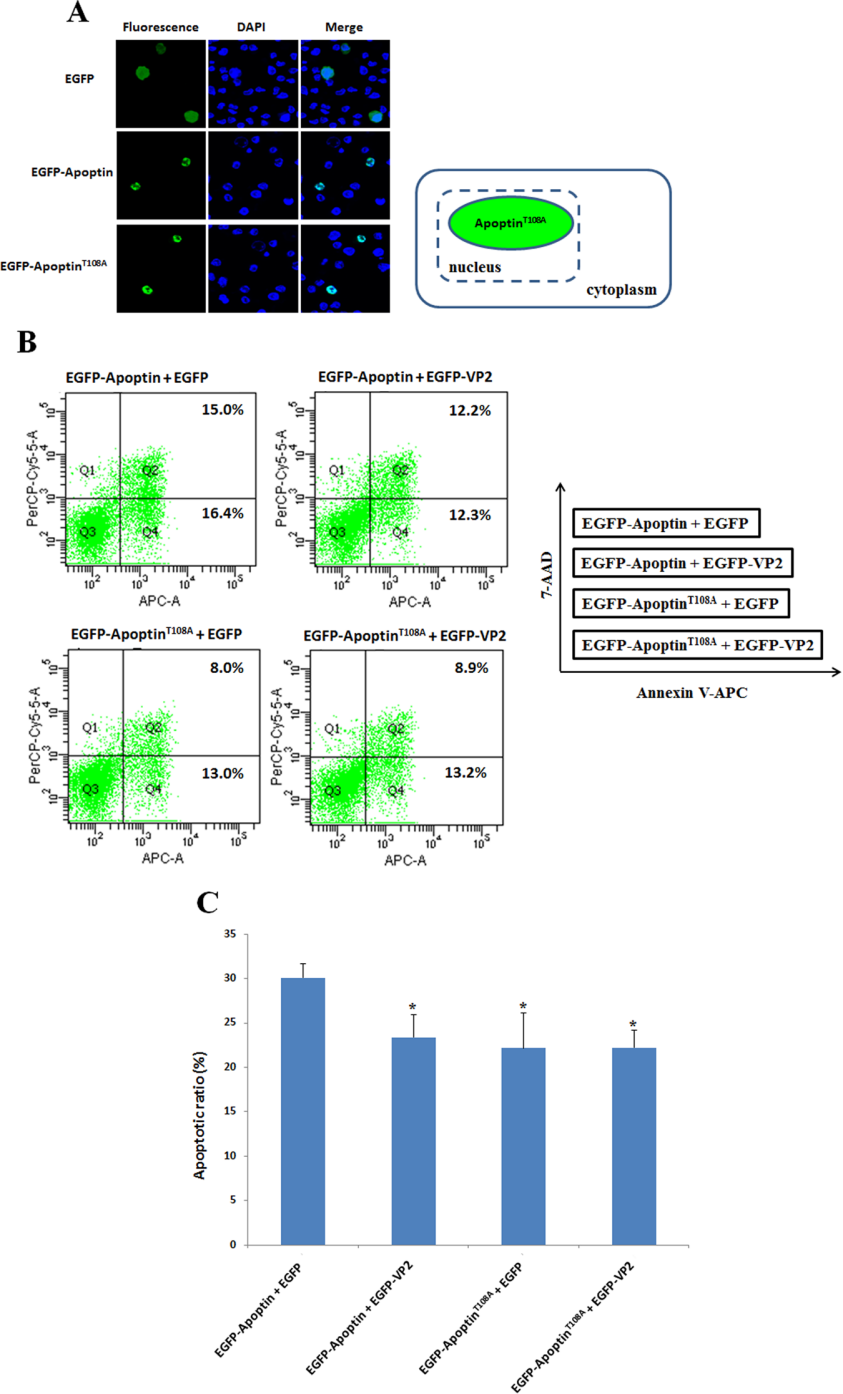
Phosphorylated Thr108 is not required for nuclear localisation but partially affects the apoptosis-inducing activity of Apoptin in MDCC-MSB1 cells. MDCC-MSB1 cells were transfected with EGFP, EGFP-Apoptin and EGFP-Apoptin^T108A^-expressing plasmids. After 48 hrs, the transfected cells were fixed and stained with DAPI to reveal the nucleus, and the subcellular localisation of the overexpressed proteins was visualised by confocal fluorescence microscopy (**A**). Corresponding dot plots of flow cytometry represent the population of apoptotic cells after co-expression of different plasmids shown in the diagram in the right panel (**B**). The relative apoptotic ratio is presented as the means ± standard deviation (**C**). A statistically significant difference vs. co-expression of EGFP-Apoptin and EGFP is observed. (*: *p* < 0.05).

Dephosphorylation of Apoptin Thr108 via VP2 suppresses apoptosis in MDCC-MSB1 cells. A previous study reported that Thr 108 phosphorylation significantly affected the subcellular localisation of this protein to the nucleus. Once Apoptin Thr108 is dephosphorylated in cancer cells, the subcellular distribution of Apoptin changes, resulting in nucleoplasmic shuffling and subsequent suppression of apoptosis^23^. Thus, to further confirm the effect of the phosphorylation status of Apoptin on apoptosis, Thr108 of Apoptin was mutated, and this protein was used to transfect MDCC-MSB1 cells for protein subcellular distribution analysis. As shown in Fig. 9B and C, mutated Thr108 was expressed in cells, and the suppression of apoptosis was demonstrated by flow cytometry analysis. The results clearly showed that the phosphorylation status of Apoptin Thr108 significantly affected the apoptosis function of this protein. Notably, dephosphorylation of Thr108 did not alter the subcellular distribution of Apoptin (Fig. 9A). As illustrated in Fig. 9A, the fluorescence emitted by mutated-Thr108 Apoptin-GFP was still observed in the nucleus of MDCC-MSB1 cells. This result suggests that apoptosis suppression is not attributable to the compartmental change of Apoptin in MDCC-MSB1 cells.

## Discussion

VP2 of CAV is a nuclear protein that harbours a nuclear localisation signal (NLS); VP2 accumulates in the nucleus when expressed in cells^25^. Apoptin also contains two NLS motifs at its C-terminus, as well as two NES motifs located at the N- and C-terminal ends of the protein^26^. Apoptin exhibits various subcellular distributions in different cells. In cancer/tumour or transformed cells, Apoptin is localised in the nucleus and frequently triggers apoptosis^22^. In contrast, apoptin is distributed in the cytosolic compartment of normal cells and does not induce apoptosis^22^. In the present study, EGFP-Apoptin was expressed in CHO-K1 cells, displaying both nuclear and cytosolic localisations (Fig. 2B). Moreover, Apoptin did not trigger apoptosis in CHO-K1 cells in the present or in previous studies^27^, a result that is consistent with the characteristics of normal versus CHO-K1 cells. When VP2 and Apoptin were co-expressed in CHO-K1 cells, VP2 was not only detected in the nucleus, but it also accumulated in the cytosolic compartment, as shown in Fig. 4A. Cheng *et al*. reported that VP2 does not contain an NES^25^, suggesting that interaction between VP2 and Apoptin might direct the export of VP2 from the nucleus to the cytosol. In the present study, further examination using *ex vivo* BiFC and *in vitro* GST pull-down assays confirmed VP2 and Apoptin interaction. In the life cycle of CAV, production of VP2 and Apoptin is abundant at 24–48 hrs post-infection^24^. Thus, during this period, VP2 and Apoptin might directly interact to form a complex to regulate viral replication or pathogenicity.

In 2009, Prasetyo *et al*. reported the disruption of Apoptin expressed in MDCC-MSB1 cells and showed that the mutation of Apoptin Thr108 could efficiently attenuate apoptosis and viral replication^28^. In the present study, interaction between VP2 and Apoptin led to a significant decrease in apoptin-mediated apoptosis via VP2 phosphatase activity through dephosphorylation of this residue of Apoptin (Figs 7 and 8). Hence, this report is the first to identify the interaction between VP2 and Apoptin and further explore the biological function. VP2 might play a key role in regulating the apoptosis-inducing activity of apoptin to maintain the productive stage of viral infection. By using a transient protein expression system, Kaffashi *et al*. showed that VP2 has apoptotic activity in MDCC-MSB1 cells^29^. However, in our study, VP2 expressed in MDCC-MSB1 cells did not exhibit apoptotic activity, even though the protein was abundantly produced in these cells (Figs 7 and 8A). As study by Kaffashi *et al*. (2015) reported no expression of VP2 in these cell^29^, a threshold expression level of VP2 might be key for the induction of apoptosis. In addition, high VP2 expression might abolish its apoptosis-inducing activity. Accordingly, there is still a need to further explore the biological function of VP2.

In a previous report, Peters *et al*. demonstrated that the subcellular distribution of Apoptin was altered from the nucleus to the nucleoplasmic compartment upon mutagenesis of residues of the catalytic site of VP2^8^. However, there is no evidence that the phosphatase activity of VP2 was decreased and that the phosphorylation status of Apoptin Thr108 was changed. In contrast, in the present study, co-expression of VP2 and Apoptin in cells indeed decreased the apoptosis triggered by Apoptin, and dephosphorylation of Thr108 of Apoptin was observed. However, dephosphorylation of Apoptin Thr108 through VP2 did not alter the subcellular distribution of Apoptin in MDCC-MSB1 cells, and mutagenesis of Thr108 did not affect the accumulation of this protein in the nucleus of MDCC-MSB1 cells, a chicken lymphoblastoid cell line.

The origin of chicken cell lines might be different from that of human cancer cells, with varying host factors. Thus, the subcellular distribution of Thr108-mutated Apoptin or the dephosphorylation of Thr108 might be further controlled by other host factors in MDCC-MSB1 cells. Notably, the apoptosis triggered by Apoptin was not completely abolished by VP2 (Fig. 8B); indeed, approximately 80% of apoptosis was maintained by Apoptin, even in the presence of VP2. As high levels of apoptosis are not conducive to the establishment of productive stages during viral replication^30^, VP2 might act a regulator to attenuate the rate of apoptosis at certain levels to promote viral propagation. In addition, these results also suggest that Thr108 is not the only amino acid controlling the apoptotic-inducing activity of Apoptin. For example, Lee *et al*. (2007) reported that despite phosphorylation of Thr108, the subcellular distribution of Apoptin in the nucleus of cancer cell lines was not affected^31^, even though dephosphorylation of Thr108 partially changed and decreased the rate of apoptosis^31^. Taken together, the results of VP2 expression in cells provided herein are partially consistent with those of Lee *et al*. In other words, VP2 might play an important role in regulating the apoptosis-inducing activity of Apoptin. With respect to the phosphorylation of Thr108, it remains unclear how many other residues contribute to Apoptin’s ability to trigger apoptosis. In 2016, Kucharski *et al*. demonstrated that all four threonine residues on Apoptin are phosphorylated by checkpoint kinase 1 (Chk1) and checkpoint kinase 2 (Chk2). When human cancer cells or MDCC-MSB1 cells were treated with inhibitors of Chk1 and Chk2, the ratio of apoptosis triggered by Apoptin showed a consequent decrease^32^. These results suggest that Thr108 is not the only residue controlling apoptosis induced by Apoptin. Notably, phosphorylation of specific residues of Apoptin has been explored in previous studies. However, there is no information regarding how these specific residues are dephosphorylated in MDCC-MSB1 or cancer cells. Nevertheless, these findings highlight the importance of VP2 in the regulation of apoptosis during CAV infection. In 2011, the first human gyrovirus (HGV), the viral genome of which is highly homologous to CAV, was discovered^33^. A small HGV-derived viral protein named HGV-Apoptin is encoded from the circular single-stranded HGV genome, with characteristics similar to those of CAV Apoptin, including the mechanism of apoptosis-inducing activity, cell cycle arrest, subcellular distribution and phosphorylation of apoptin residues in cancer cells^34–37^. Based on previous reports, participation of CAV VP2 in the apoptosis induced by CAV or HGV Apoptin might be useful for comprehensively reexamining the biological role of VP2 or investigating the effect of VP2 on Apoptin-induced apoptosis. Moreover, some Apoptin-interacting molecules such as peptidyl-prolyl isomerase like 3 (Ppil3)^38^, huntingtin-interacting protein 1 (Hip1)^39^, anaphase-promoting complex 1 (APC1)^40^ or death effector domain-associated factor (DEDAF)^41^ identified in Apoptin-induced apoptotic cells might be examined while investigating the importance of VP2 in the regulation of apoptosis when Apoptin is introduced into cancer cells or in CAV-infected host cells. Although numerous anti-cancer therapeutic agents have been reported by many research groups, cancer reoccurrence is a frequently encountered problem after therapy. Some reasons for the failures of anti-cancer treatment are the presence of resistance to anti-cancer drugs and the initiation of cancer stem cells (CSC)^42,43^. Comparative studies of normal stem cells and CSCs are on-going. Apoptin or the regulation of CAV Apoptin by VP2 might be applicable for the treatment of CSCs in further exploitation of anti-cancer strategies.

In conclusion, this study is first to describe the interaction between VP2 and Apoptin of CAV. When VP2 directly interacts with Apoptin in MDCC-MSB1 cells, the rate of apoptosis triggered by Apoptin was attenuated by VP2 via dephosphorylation of Apoptin Thr108.

## Methods

### Cell culture

Chinese Hamster Ovary (CHO-K1) cells were purchased from Bioresource Collection and Research Center (BCRC 6006) in Taiwan, and chicken lymphoblastoid (MDCC-MSB1) cells were purchased from CLS Cell Lines Service GmbH in Germany. CHO-K1 and MDCC-MSB1 cells were respectively maintained in Ham’s F12 medium (HyClone, USA) and RPMI 1640 medium (HyClone, USA) supplemented with 10% foetal bovine serum (FBS; HyClone, USA) and 1% P/S (Penicillin/Streptomycin solution) (Gibco, USA). Prior to these experiments, CHO-K1 cells were cultured in 10-cm culture dishes, and MDCC-MSB1 cells were maintained in T75 flasks at 37 °C in a humidified cell culture incubator with 5% CO_2_.

### Construction of expression plasmids for the BiFC assay and subcellular co-localisation studies

The plasmids used for the bimolecular fluorescence complementation assay were constructed as described below. For YFP fragments, residues 1–154 (YN) and 155–238 (YC), which associate to form a biomolecular fluorescent complex^44^, were selected as the two YFP fragments for complementation in BiFC assays. First, the sequence encoding the N-terminus and C-terminus of enhanced (E)YFP were polymerase chain reaction (PCR) amplified using the specific primers EYFPN-f and EYFPN-r (YN) or EYFPC-f and EYFPC-r (YC), as listed in Table 1. The forward primers, EYFPN-f and EYFPC-f, contained linker sequences encoding RSIAT (for YN) and RPACKIPNDLKQKVMNH (for YC) as well as a recognition site for *Xho* I at the 5′-end; the reverse primers EYFPN-r and EYFPC-r incorporated stop codons and the recognition site for *Xba* I (Table 1). After subcloning the amplified YN and YC fragments into the expression vector pcDNA3.1 (#V80020, Invitrogen, USA) using the cloning sites *Xho* I/*Xba* I, the resulting plasmids were named pYNc and pYCc according to the schematic representation in Fig. 1, showing plasmids containing the N- and C-terminal fragments of the EYFP gene, respectively. In the next step, the full-length cDNAs of the CAV VP2 and VP3 genes were PCR amplified using specific primer sets, wt-VP2-f and wt-VP2-r for VP2 and wt-VP3-f and wt-VP3-r for VP3, to introduce the restriction sites *EcoR* I/*Xho* I. The amplified genes VP2 and VP3 were subcloned into previously constructed pYNc and pYCc to generate plasmids pYNc-VP2 and pYCc-VP3, respectively (also shown in Fig. 1). The resulting constructs were subsequently used to transfect CHO-K1 cells according to the transfection procedure for the BiFC assay.

**Table 1:**
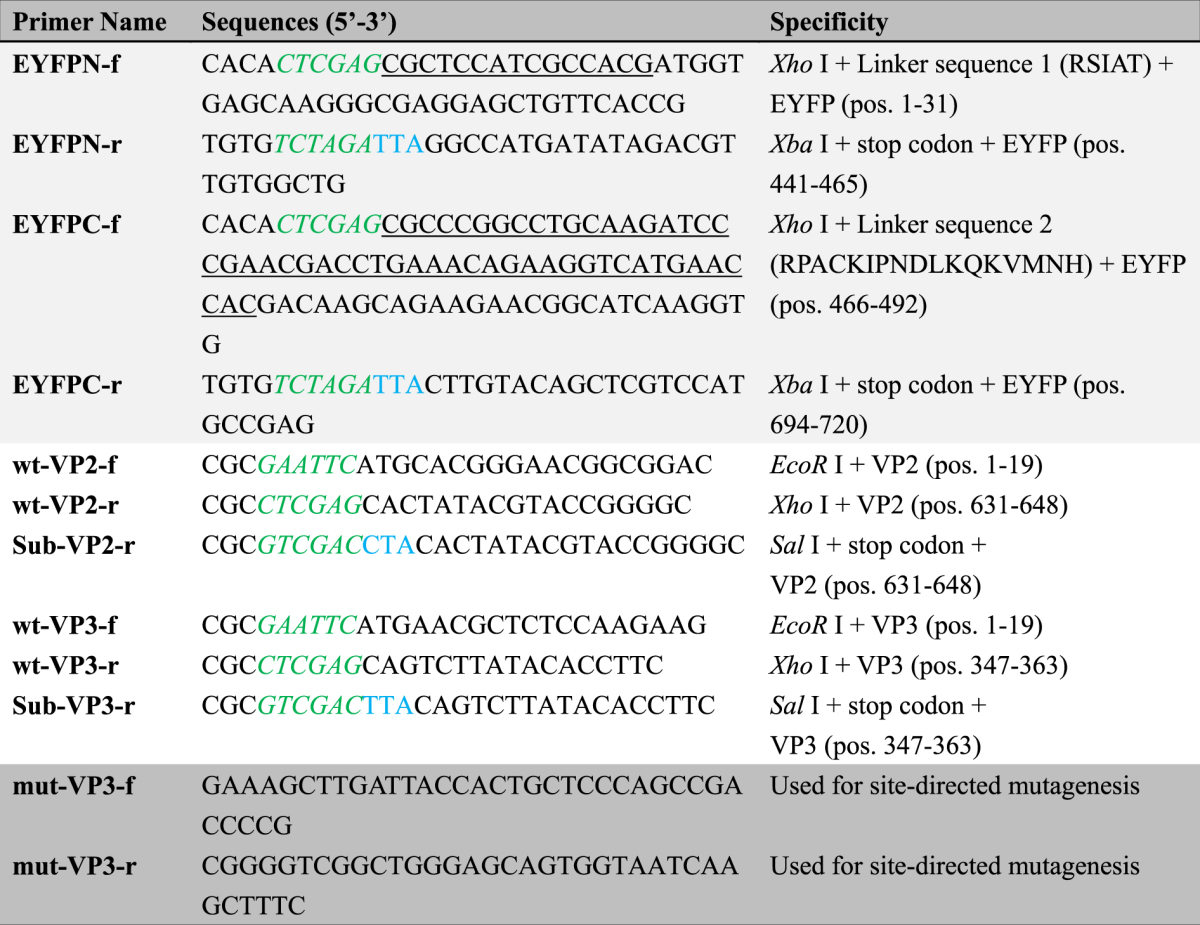
Primers used for construction of expression plasmids in this work. Primers sequences contained extra recognition sites at the 5′-end. “*pos*.” represents specific sequence positions of the target gene.

For subcellular co-localisation studies, gene fragments containing the entire open reading frames of CAV VP2 and VP3 were PCR amplified using other primer sets, wt-VP2-f and Sub-VP2-r and wt-VP3-f and Sub-VP3-r, respectively. The amplified VP2 and VP3 gene fragments were next incorporated into the corresponding *EcoR* I/*Sal* I sites of the pmCherry-C1 (#632524, Clontech, USA) and pEGFP-C2 (#6083-1, Clontech, USA) expression vectors to generate the recombinant plasmids pmCherry-VP2 and pEGFP-VP3, respectively (see Fig. 1). After transfection of the above plasmids, fluorescent protein-fused VP2 and Apoptin were expressed carrying mCherry and EGFP as reporters for the detection of subcellular localisation.

Mutation of Apoptin Thr108 was achieved through site-directed mutagenesis using the QuikChange II Site-directed mutagenesis kit (Agilent, USA) according the manufacturer’s instructions with pEGFP-VP3 as the plasmid template; the two complementary mutagenic primers used for mutagenesis were mut-VP3-f and mut-VP3-r, as shown in Table 1. The mutated constructs were transfected into MDCC-MSB1 to express EGFP-Apoptin^T108A^ to assess the effects of Thr108 on the subcellular localisation and apoptosis-inducing ability of Apoptin.

### Cell transfection of CHO-K1 cells

Prior to transfection, CHO-K1 cells were plated at a density of 2.4 × 10^5^ cells/well in 6-well plates with 2.5 ml complete growth medium per well and incubated overnight. For transfection, 2 μg of plasmids and 4 μl X-tremeGene HP DNA transfection reagent (Sigma, USA) in 0.25 ml serum-free Opti-MEM medium (Gibco, USA) were gently pipetted into the mixture, which was incubated at room temperature for 20 min. The resulting transfection mixture was added dropwise to the CHO-K1 culture, and the cells were grown in an incubator for 24 to 48 hrs. At the end of transfection, the cells were examined by confocal microscopy.

### Transfection of MDCC-MSB1 cells

MDCC-MSB1 cells in log phase were collected and washed twice in serum-free RPMI 1640 medium, and 4 × 10^6^ cells were resuspended in 400 μl RPMI 1640 containing 15 μg of plasmid DNA in an Eppendorf tube. After gentle pipet-mixing, the cells-DNA mixture was transferred to a 0.4-cm gap electroporation cuvette, which was incubated on ice for 5 min prior to transfection. Transfection was performed using Time Constant Protocol of Gene Pulser II (Bio-Rad, USA) at a 300-V operating voltage with a time constant of 34 milliseconds. After electroporation, the cells were incubated at room temperature for 10 min and subsequently cultured in 3 ml of complete medium in a 6-well plate for 48 hrs. At the end of the transfection, the cells were observed by confocal microscopy or lysed to isolate proteins for western blot analysis.

### Sample preparation and confocal microscopic observation

CHO-K1 and MDCC-MSB1 cells were transfected with recombinant plasmids, and images were captured by confocal fluorescence microscopy to verify transfected and non-transfected cells based on protein fluorescence. The cell culture samples were mounted onto a glass slide using Gelvatol mounting medium (Sigma, USA). Confocal laser scanning microscope (CLSM) images were obtained using a Leica TCS SP8 confocal microscope, and the images were integrated using LAS X Leica Confocal Software. EGFP fluorescence was observed through excitation at 488 nm, and mCherry fluorescence was observed through excitation at 568 nm. In the BiFC assay, reassembly of EYFP fluorescence was observed through excitation at 514 nm. For DAPI staining, cells were fixed in the dark with 4% formaldehyde and incubated for 30 min at room temperature. After removing the formaldehyde, the cells were incubated in 0.1% phosphate-buffered saline-Tween 20 (PBS-T) with 1 μg/ml DAPI for 5 min at 37 °C in the dark. DAPI emits blue fluorescence upon binding to nuclear DNA through excitation by UV light.

### Constructs for protein expression, bacterial strain and its inoculation

For expression of recombinant VP2 and Apoptin, the plasmids pVP2 and pVP3_opt_, constructed in a previous work, were respectively used to transform *Escherichia coli* strain BL21 (DE3)-*pLys*S^45^. These two plasmids were constructed using pGEX-4T-1, a plasmid harbouring the entire CAV VP2 and VP3 genes, which were alternatively named pGEX-VP2 and pGEX-VP3_opt_ in the present study, as illustrated in Fig. 1. With respect to Trx-H-VP2 and Trx-H-Apoptin, two other constructs, pET32a-VP2 and pET32a-VP3_opt_ harbouring the full-length cDNAs of the CAV VP2 and VP3 genes, respectively (see also Fig. 1), were subcloned from the plasmids pGEX-VP2 and pGEX-VP3_opt_ into vector pET32a using the cloning sites *EcoR* I and *Xho* I. These two constructs were also used to transform BL21 (DE3)-*pLys*S strains for protein expression. The generation of competent cells as well as inoculation and induction were performed using 10 ml of LB medium in 50-ml flasks by culturing at 37 °C according to Lai *et al*.^45^.

### Purification of Trx-H-VP2 and Trx-H-Apoptin using an Ni-NTA affinity column

After induction with isopropyl β-D-1-thiogalactopyranoside (IPTG), *E. coli* cells BL21(DE3)-*pLys*S harbouring pET32a-VP2 and pET32a-VP3_opt_ were harvested from LB medium, disrupted and prepared according to Lee *et al*.^46^. The resulting cell supernatant was loaded onto Ni-NTA agarose (Invitrogen, CA, USA) packed into an Enco-column (Bio-Rad, USA). The purification procedure was also performed according to Lee *et al*.^46^. The concentration of purified protein was determined using a Micro BCA kit (Pierce, Rockford, IL), and the protein sample was analysed by 12.5% sodium dodecyl sulphate polyacrylamide gel electrophoresis (SDS-PAGE) and western blotting with appropriate antibodies.

### *In vitro* GST pull-down assay

An *in vitro* GST pull-down assay was used to confirm protein-protein interaction between VP2 and Apoptin*. E. coli*-expressed GST-VP2 and GST-Apoptin were each used as bait with the prey Trx-H-VP2 and Trx-H-Apoptin. To assess the occurrence of an interaction between GST-VP2 and Trx-H-Apoptin or GST-apoptin and Trx-H-VP2, Trx-H-VP2 and Trx-H-Apoptin were co-purified with GST-Apoptin and GST-VP2, respectively, using a GST sepharose 4B FF column (GE Healthcare, USA). The GST fusion protein was purified according to Lee *et al*.^47^. After binding and washing the GST Sepharose 4B FF column, 300 μg of Ni-NTA-purified Trx-H-VP2 and Trx-H-Apoptin was respectively reloaded onto the same column to reperform the entire the purification procedure, including binding and washing. Next, the proteins were eluted using 30 μl elution buffer (50 mM Tris-HCl, pH 8.0, 10 mM dithiothreitol (DTT), and 10 mM reduced glutathione). The resulting proteins were further examined by SDS-PAGE and western blot analysis.

### Determination of apoptosis using flow cytometry

Transfected MDCC-MSB1 cells were washed three times with PBS. A total of 1 × 10^6^ cells were collected and suspended in Annexin-V binding buffer (BD Bioscience, USA) and treated with 7-amino-actinomycin (7-AAD) and Annexin-V conjugated to allophycocyanin (APC) for labelling. The population of cells emitting GFP fluorescent corresponding to successfully transfected MSB1 cells were gated and analysed using a BD FACSCanto II (BD Bioscience, USA). A total of 10,000 GFP-expressing cells were acquired, and apoptosis was further determined by flow cytometry.

### Western blot analysis

Transfected cells were collected and washed twice with PBS and subsequently directly lysed in 1X RIPA buffer (50 mM Tris-HCl, pH 7.5, 150 mM NaCl, 1 mM ethylenediaminetetraacetic acid (EDTA), and 1% Triton X-100) containing 1X protease inhibitor cocktail (Roche, USA) and 1X phosphatase inhibitor cocktail (Roche, USA). After performing SDS-PAGE and transferring the proteins to polyvinylidene fluoride (PVDF) membranes (Millipore, USA), the membranes were incubated with anti-VP2, anti-Apoptin, and anti-108p-Apoptin primary antibodies (GeneMark Company in Taiwan); commercial primary antibodies anti-GST (Novus, USA) and anti-6xHis (Novus, USA); and secondary antibodies anti-mouse and anti-rabbit linked horseradish peroxidase (Jackson, USA). The membranes were developed using an enhanced chemiluminescence (ECL) system (ThermoFisher, USA).

### Statistical analysis

The data are presented as the means ± standard error of the mean (SEM) of three independent experiments (n = 3). Statistical calculations were performed using one-way analysis of variance (ANOVA) to display differences between groups. A *p*-value < 0.05 was considered statistically significant.

## Electronic supplementary material

Supplementary Information
