# Supplementary material for: VP2 of Chicken Anaemia Virus Interacts with Apoptin for Down-regulation of Apoptosis through De-phosphorylated Threonine 108 on Apoptin
Source: Sci Rep. 2017 Nov 1;7:14799. doi: 10.1038/s41598-017-14558-8 (PMC5665943; doi:10.1038/s41598-017-14558-8)
Supplement: Supplementary file 1 — Supplementary Information [file 41598_2017_14558_MOESM1_ESM.pdf]

VP2 of Chicken Anaemia Virus Interacts with Apoptin for Down-regulation of Apoptosis through De-phosphorylated Threonine 108 on Apoptin

Guan-Hua Lai[1Φ], Yi-Yang Lien [2Φ], Ming-Kuem Lin [3], Jai-Hong Cheng[4], Jason TC Tzen[1], Fang-Chun Sun[5], Meng-Shiunn Lee[6], Hsi-Jien Chen[7], Meng-Shiou Lee[3]*

[1]Graduate Institute of Biotechnology, National Chung Hsing University, Taichung 40402, Taiwan.
[2] Department of Veterinary Medicine, National Pingtung University of Science and Technology, Pingtung, Taiwan
[3]Department of Chinese Pharmaceutical Science and Chinese Medicine Resources, China Medical University, Taichung, Taiwan..
[4]Center for Shockwave Medicine and Tissue Engineering, Department of Medical Research, Kaohsiung Chang Gung Memorial Hospital and Chang Gung University College of Medicine, Kaohsiung, Taiwan.
[5] Department of Bioresources, Da-Yeh University, Changhua, Taiwan.
[6] Research Assistance Center, Show Chwan Memorial Hospital, Changhua, Taiwan.
[7] Department of Safety, Health and Environmental Engineering, Ming Chi University of Technology, New Taipei, Taiwan. E-mail: leemengshiou@mail.cmu.edu.tw

[Φ] These authors contributed equally to this study.

*Corresponding author:
Department of Chinese Pharmaceutical Science and Chinese Medicine Resources, China Medical University, 91, Hsueh-Shih Road, Taichung, Taiwan.
Fax: +886-4-24075683
Tel.: +886-4-2205-3366 ext 5208
E-mail address: leemengshiou@mail.cmu.edu.tw (Lee, M-S.)

Supplementary Fig. 1A

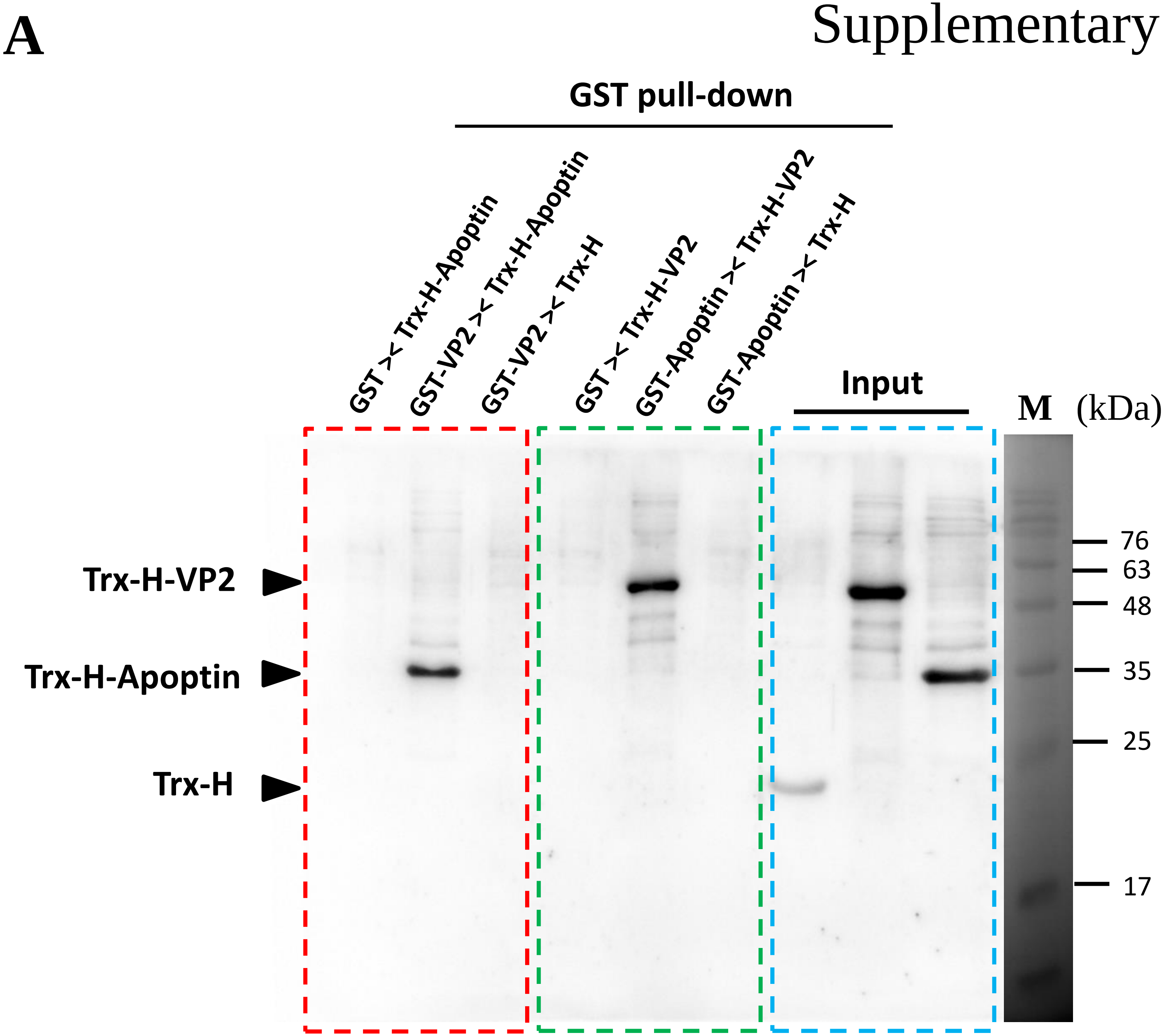

Supplementary Fig. 1B

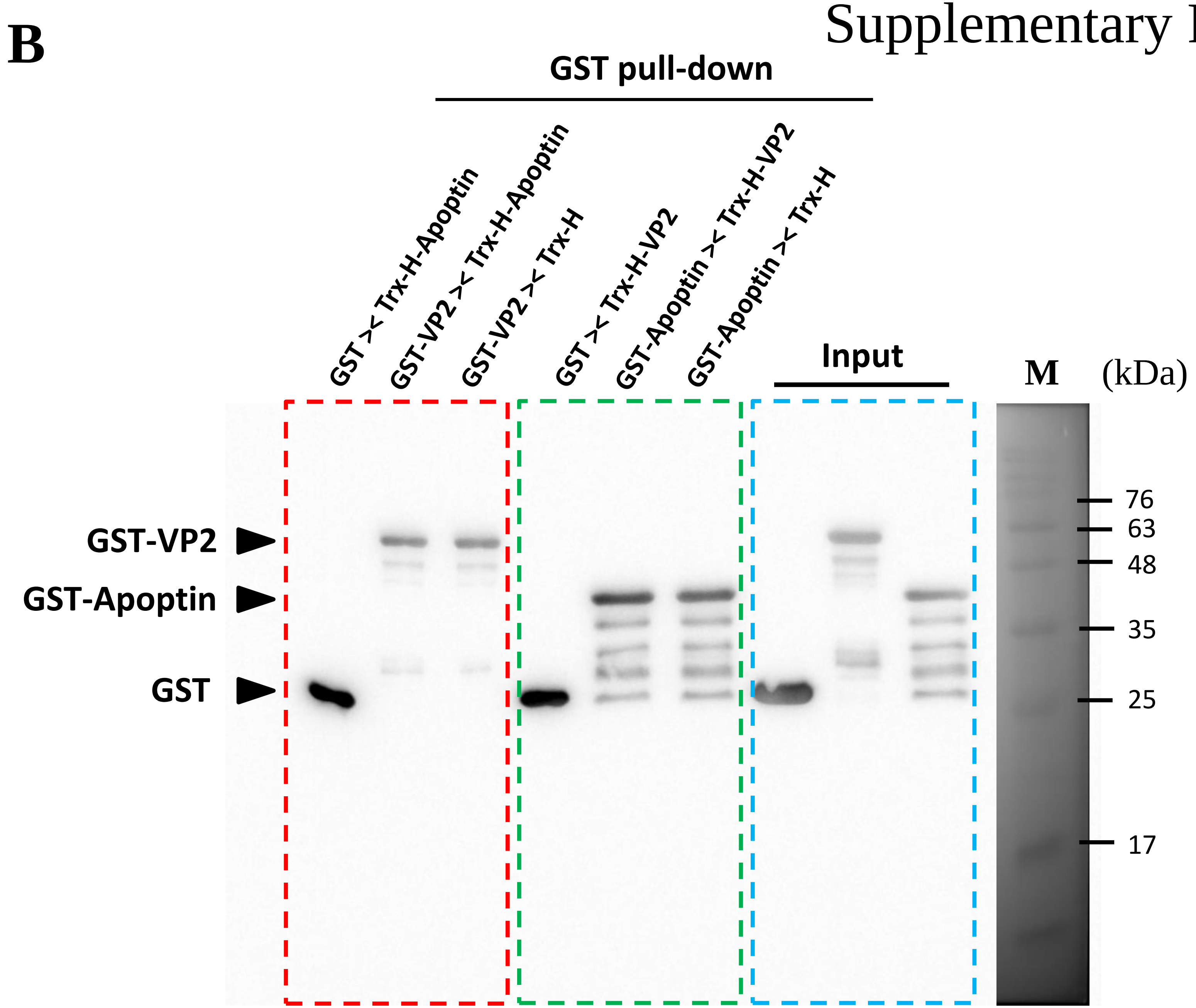

Supplementary figure 1. Original western blotting image of figure 6. The top and bottom middle panels of figure 6 were cropped and edited from the red box of the original images **(A)** and **(B)**, respectively. The top and bottom right panels in figure 6 were obtained from the green boxes of the original images **(A)** and **(B)**, respectively. The input panel (top and bottom left) in figure 6 was captured together from the blue boxes of the original images **(A)** and **(B)**. Images **(A)** were obtained from western blot assay with the α-6xHis monoclonal antibody; images **(B)** indicate the western blot assay performing using the α-GST monoclonal antibody. The eluted fractions collected from GST pull-down assay are subtitled in the original images, and the arrows indicate the blotting signals corresponding to recombinant proteins. Lane M, pre-stained protein ladder marker.

Supplementary Fig. 2A

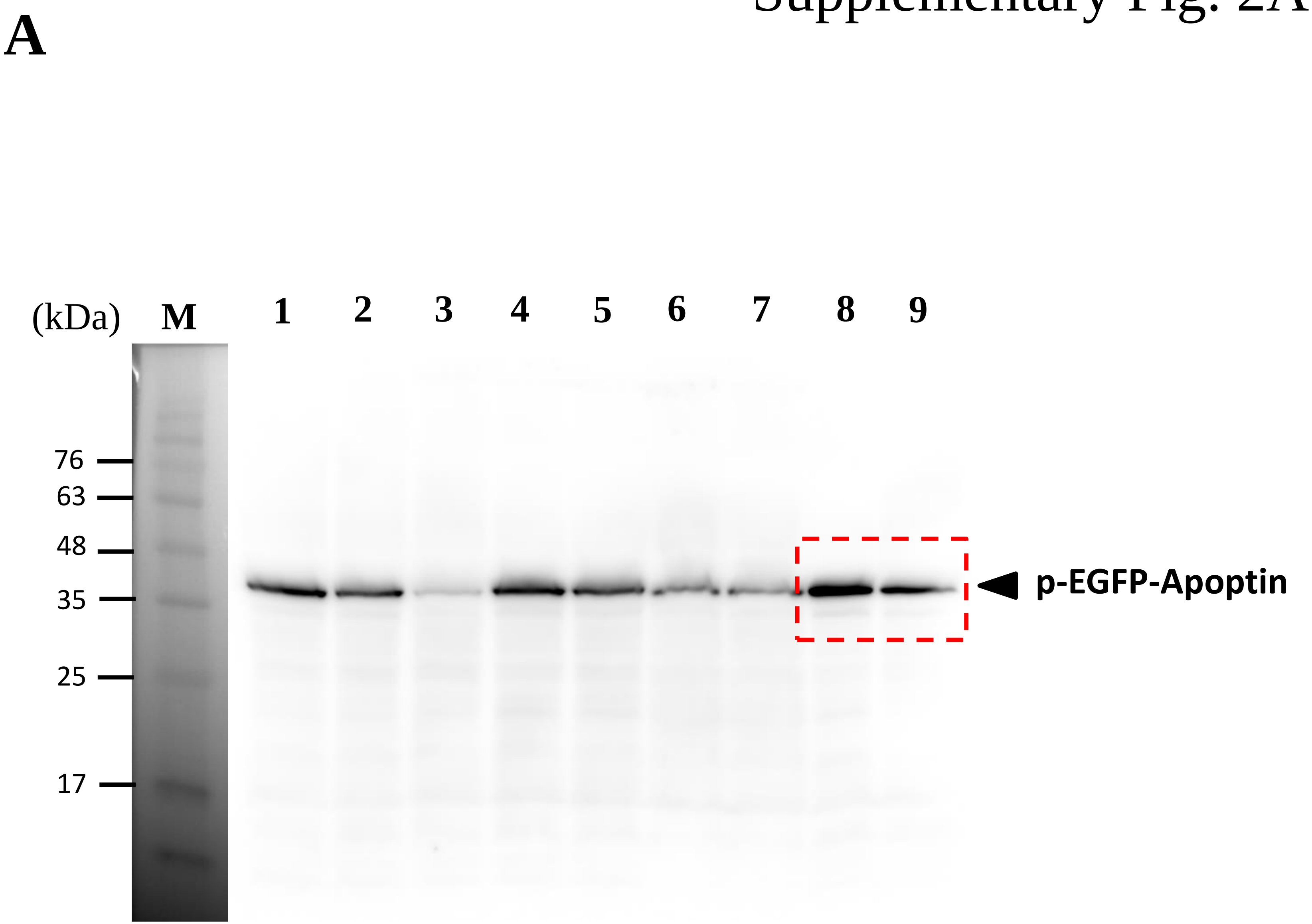

Supplementary Fig. 2B

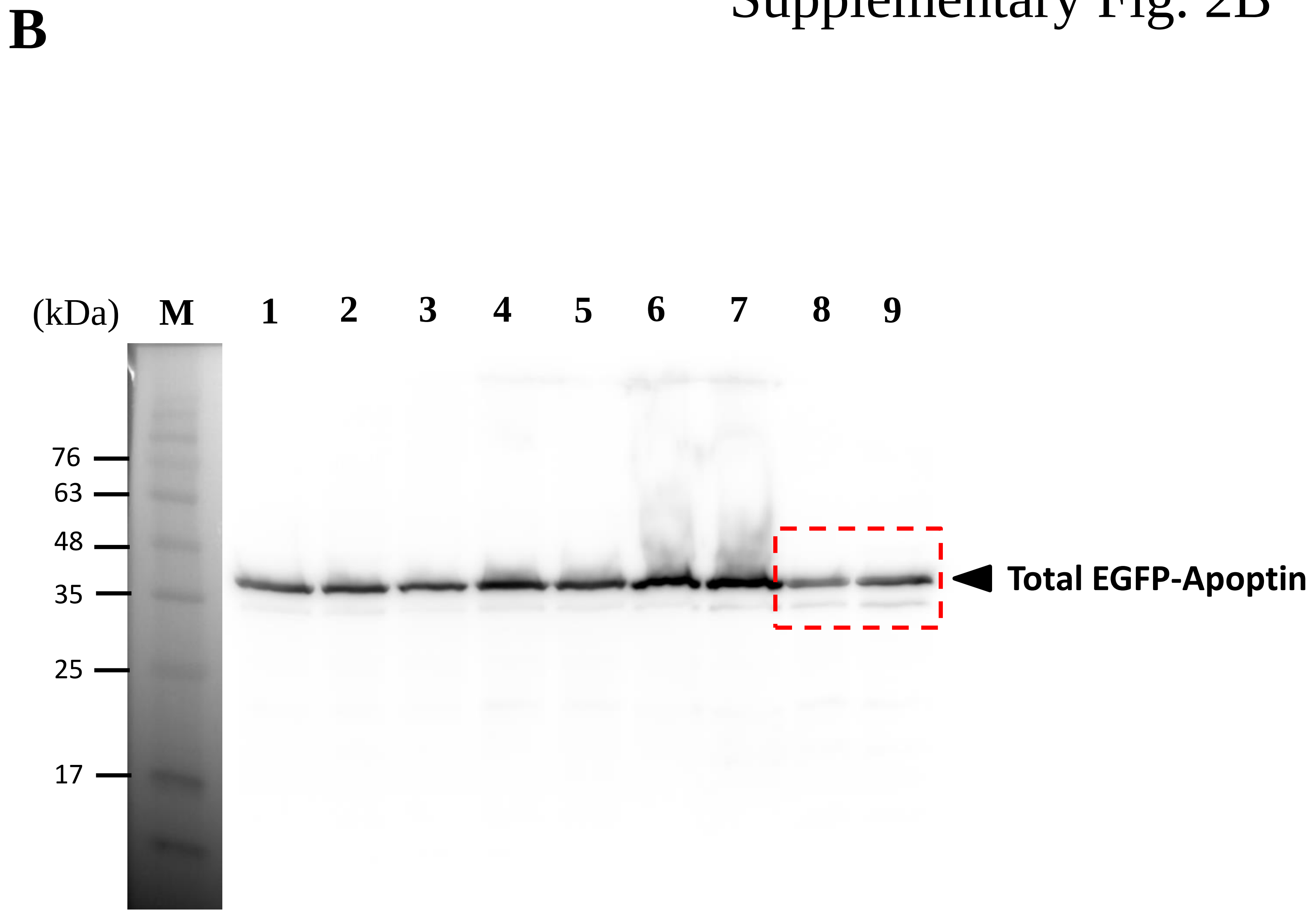

Supplementary Fig. 2C

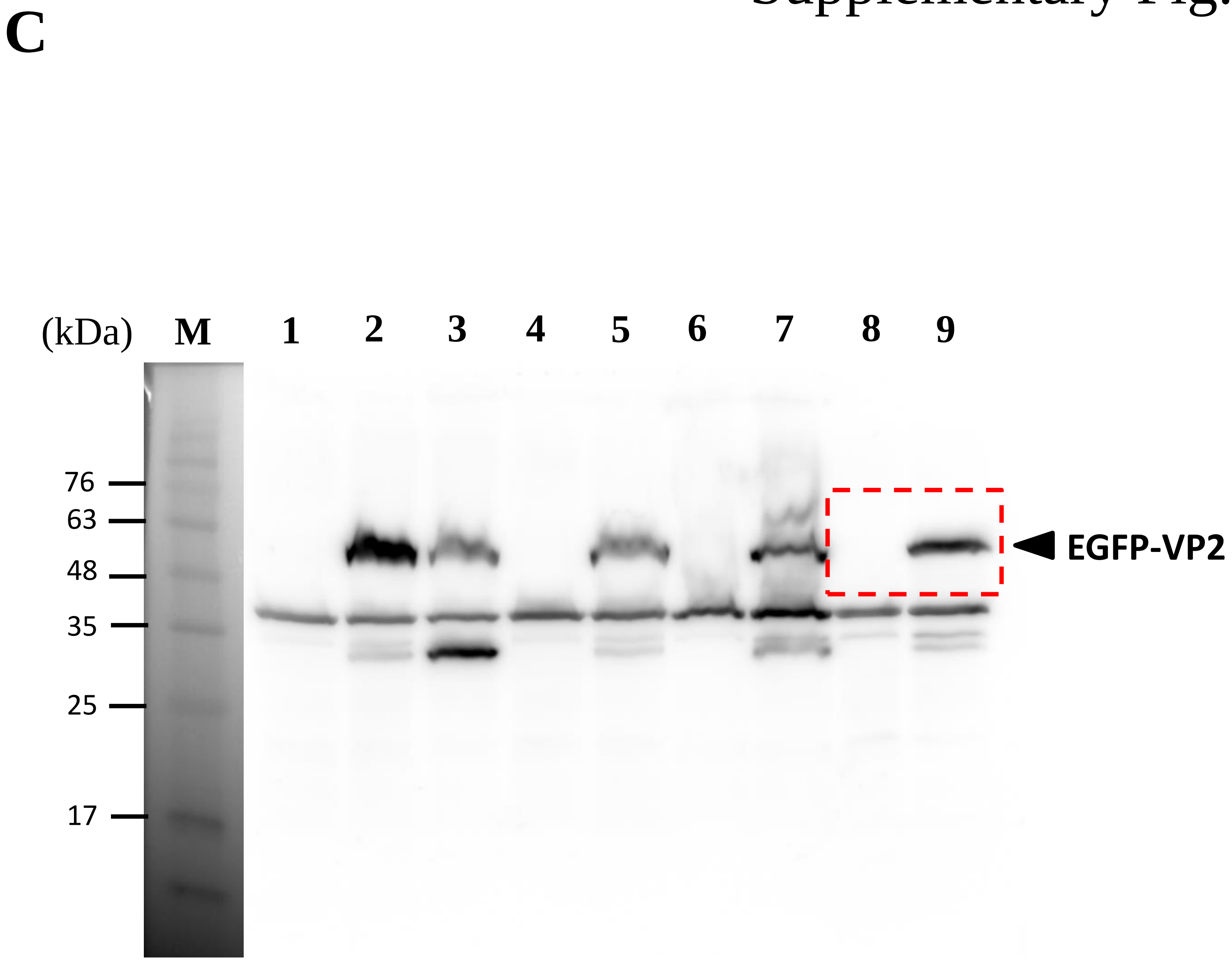

Supplementary figure 2. Original western blotting image of figure 8. The protein-transferred PVDF membrane was used for western blotting with an anti-phosphorylated apoptin threonine 108 antibody **(A)**. This western blot (WB) membrane was stripped and then re-stained with other primary antibodies: α-apoptin **(B)** and α-VP2 **(C)** sequentially. The top, middle and bottom panels of figure 8A were obtained from the red boxes of the original images **(A)**, **(B)** and **(C)**, respectively. Lane M, pre-stained protein ladder marker. Lanes 1, 4, 6, and 8 show the WB results of transfected cells expressing EGFP-apoptin; Lanes 2, 3, 5, 7, and 9 indicate the WB results of transfected cells co-expressing EGFP-apoptin and EGFP-VP2 simultaneously. The arrow indicates the blotting signals corresponding to overexpressed proteins. The intensities of all signals in the lanes were manipulated for statistical analysis using triplicate individual experimental results; for example, group 1 of lane 1 and land 2, group 2 of lane 4 and lane 5, and group 3 of lane 8 and lane 9 were used to determine the relative phosphorylation level of threonine 108 of apoptin (108p-Apoptin) for normalisation, respectively. The result of normalisation is presented as **figure 8B**.
